# Accessory subunit NDUFB4 participates in mitochondrial complex I supercomplex formation

**DOI:** 10.1016/j.jbc.2024.105626

**Published:** 2024-01-09

**Authors:** Gaganvir Parmar, Claire Fong-McMaster, Chantal A. Pileggi, David A. Patten, Alexanne Cuillerier, Stephanie Myers, Ying Wang, Siegfried Hekimi, Miroslava Cuperlovic-Culf, Mary-Ellen Harper

**Affiliations:** 1 Department of Biochemistry, Microbiology and Immunology, Faculty of Medicine, University of Ottawa, Ottawa, Ontario, Canada; 2 Ottawa Institute of Systems Biology, University of Ottawa, Ontario, Canada; 3 Children’s Hospital of Eastern Ontario Research Institute, Ottawa, Ontario, Canada; 4 Department of Biology, McGill University, Montreal, Quebec, Canada; 5 National Research Council of Canada, Digital Technologies Research Centre, Ottawa, Ontario, Canada

**Keywords:** mitochondria, electron transport chain, supercomplexes, NDUFB4, oxidative phosphorylation, respirasome, steady-state metabolomics

## Abstract

Mitochondrial electron transport chain complexes organize into supramolecular structures called respiratory supercomplexes (SCs). The role of respiratory SCs remains largely unconfirmed despite evidence supporting their necessity for mitochondrial respiratory function. The mechanisms underlying the formation of the I_1_III_2_IV_1_ “respirasome” SC are also not fully understood, further limiting insights into these processes in physiology and diseases, including neurodegeneration and metabolic syndromes. NDUFB4 is a complex I accessory subunit that contains residues that interact with the subunit UQCRC1 from complex III, suggesting that NDUFB4 is integral for I_1_III_2_IV_1_ respirasome integrity. Here, we introduced specific point mutations to Asn24 (N24) and Arg30 (R30) residues on NDUFB4 to decipher the role of I_1_III_2_-containing respiratory SCs in cellular metabolism while minimizing the functional consequences to complex I assembly. Our results demonstrate that NDUFB4 point mutations N24A and R30A impair I_1_III_2_IV_1_ respirasome assembly and reduce mitochondrial respiratory flux. Steady-state metabolomics also revealed a global decrease in citric acid cycle metabolites, affecting NADH-generating substrates. Taken together, our findings highlight an integral role of NDUFB4 in respirasome assembly and demonstrate the functional significance of SCs in regulating mammalian cell bioenergetics.

Mitochondrial energy transduction is achieved through oxidative phosphorylation (OXPHOS) *via* the electron transport chain (ETC). Electrons are transferred by ETC enzyme complexes (C) I-IV and the electron carriers, coenzyme Q (CoQ), and cytochrome *c* (cyt c), to molecular oxygen (O_2_) through a series of redox reactions. The free energy generated from the flow of electrons drives matrix proton extrusion at CI, CIII, and CIV to create a proton gradient across the mitochondrial inner membrane (MIM) that drives the activity of the F_1_F_o_-ATP synthase to phosphorylate ADP to ATP.

Mitochondrial protein complexes CI (NADH-ubiquinone oxidoreductase), CIII (CoQ-cyt c reductase), and CIV (cyt c oxidase) can assemble into higher order structures (*e.g.*, I_1_III_2_, III_2_IV_1_, and I_1_III_2_IV_1_) called respiratory supercomplexes (SCs) (1) that retain enzymatic and respiration activities (2, 3). Respiratory SCs that contain CI, CIII, and CIV (I_1_III_2_IV_1_) have been termed “respirasomes” and can catalyze the redox reactions for electron transfer of NADH at CI to O_2_ at CIV (1). While CII (succinate dehydrogenase [SDH]) is not empirically detected in mammalian respiratory SCs (4), ubiquinol produced by CII can be processed by the III_2_IV_1_ respiratory SC. Moreover, structural studies indicate that CII may form weak protein–protein interactions with highly oligomerized forms of the I_2_III_2_IV_2_ respirasome, termed megacomplexes (5, 6, 7). While the functional implications of respiratory SCs are still actively debated (8), the assembly of respiratory SCs is integral for CI assembly and stability (9, 10, 11). Respiratory SCs have also been proposed to limit mitochondrial ROS production (12, 13) and optimize the kinetics of electron transfer by decreasing diffusion distances (14).

The “plasticity” model proposes that respiratory enzymes exist as free preassembled individual complexes, which can undergo dynamic rearrangement into SCs in response to various stressors to meet cellular metabolic requirements (15, 16, 17). However, this model has recently been challenged by the “cooperative assembly model,” where CI, CIII, and CIV precursor subunits can assemble as part of a respiratory SC before the addition of catalytic modules to complete the assembly of the individual complexes. This model is supported by evidence of partially assembled respirasome subcomplexes that contain variations of pre-CI modules, pre-CIII_2_, and CIV (18). The interactions between CI and CIII are of particular importance, considering that the majority of mammalian CI (∼80%+) is incorporated into SCs and that the I_1_III_2_ respiratory SC is conserved across mammalian species (11, 19). Mammalian CI is comprised of 45 protein subunits in total that are distributed into a matrix arm containing the N- and Q-modules, and a membrane arm containing the proton translocation module (P module) with proximal (P_p_) and distal (P_D_) segments further subdivided into P_p-a_, P_p-b_, P_D-a_, and P_D-b_ submodules (20, 21). The N- and Q-modules contain 14 subunits that are directly involved in catalytic activity (22). The remaining subunits (termed “accessory”) are likely involved in supporting the structure of the redox centers, maintaining the integrity of the complex (23, 24, 25). Most CI accessory subunit KOs do not display CI-containing SCs at a steady state, suggesting that accessory subunits are integral to CI assembly and stability, as well as respiratory SC formation (25).

Structural studies of the mammalian I_1_III_2_IV_1_ respirasome have revealed that transmembrane complex I accessory subunits located on the membrane arm (P-modules) constitute the CI-CIII_2_ interface and interact with CIII subunits (26, 27, 28). Specifically, NDUFB4 and NDUFB9, both located on the distal P_D_-module, interact with the UQCRFS1 (the Rieske protein) and a highly conserved loop of UQCRC1 in the mitochondrial matrix (7, 27, 28). NDUFA11, located on the proximal half of the P-module (P_P_-module), interacts with UQCRB and UQCRQ, and along with NDUFB4, also interacts with UQCRQ (23, 27, 28). Moreover, the N termini of NDUFB4 and NDUFB8 contact UQCRC10.

The discovery of the SC assembly factor 1 (SCAF1/COX7A2L) and its role in III_2_IV_1_ SC assembly has prompted a search for additional protein factors regulating respiratory SCs (29). However, in models where SCAF1 is shown to be required for III_2_IV_1_ SC formation, the levels of the I_1_III_2_IV_1_ respirasome SC remain unaffected by ΔSCAF1 (30), suggesting that additional factors can independently maintain interactions between CI and CIII_2_ or CIV to mediate respirasome formation (31, 32). Other regulatory protein factors that participate in the assembly and stabilization of respiratory SCs have been identified, including members of the hypoxia-inducible domain family and the ubiquinol-cyt c reductase complex assembly factor 3 (C11orf83/UQCC3) (33, 34, 35, 36). However, knowledge of protein factors specific to CI-containing SCs, such as the I_1_III_2_IV_1_ respirasome, remains limited to date.

Considering that SCAF1/COX7A2L is a structural subunit of the III_2_IV_1_ SC, we hypothesized that complex I accessory subunits suggested to interact with CIII_2_ in assembled SCs may also participate in the assembly of CI-containing SCs, such as the I_1_III_2_IV_1_ respirasome. To address this, we explored respiratory SC formation through the noncatalytic CI subunit NDUFB4, which is hypothesized to interact with residues of the CIII subunit UQCRC1 *via* hydrogen bonds (27). By introducing point mutations at Asn24 and Arg30 of NDUFB4, our results demonstrate that NDUFB4 participates in respirasome formation and alters cellular bioenergetics and metabolism.

## Results

### N24A and R30A mutations of NDUFB4 modify respiratory SC formation

Evidence from *in vitro* studies indicates that subassemblies of the P_D-a_ module may act as a scaffold to initiate conglomeration with CIII_2_ and CIV to form respiratory SCs (18). Structural studies have identified key protein–protein interactions that occur between transmembrane complex I accessory subunits (NDUFA11, NDUFB4/8/9) located in the P_D_ and P_P_ modules of the CI membrane arm that interact with specific subunits on CIII_2_ and therefore may participate in respiratory SC formation (18, 26, 27, 28).

**Figure 1:**
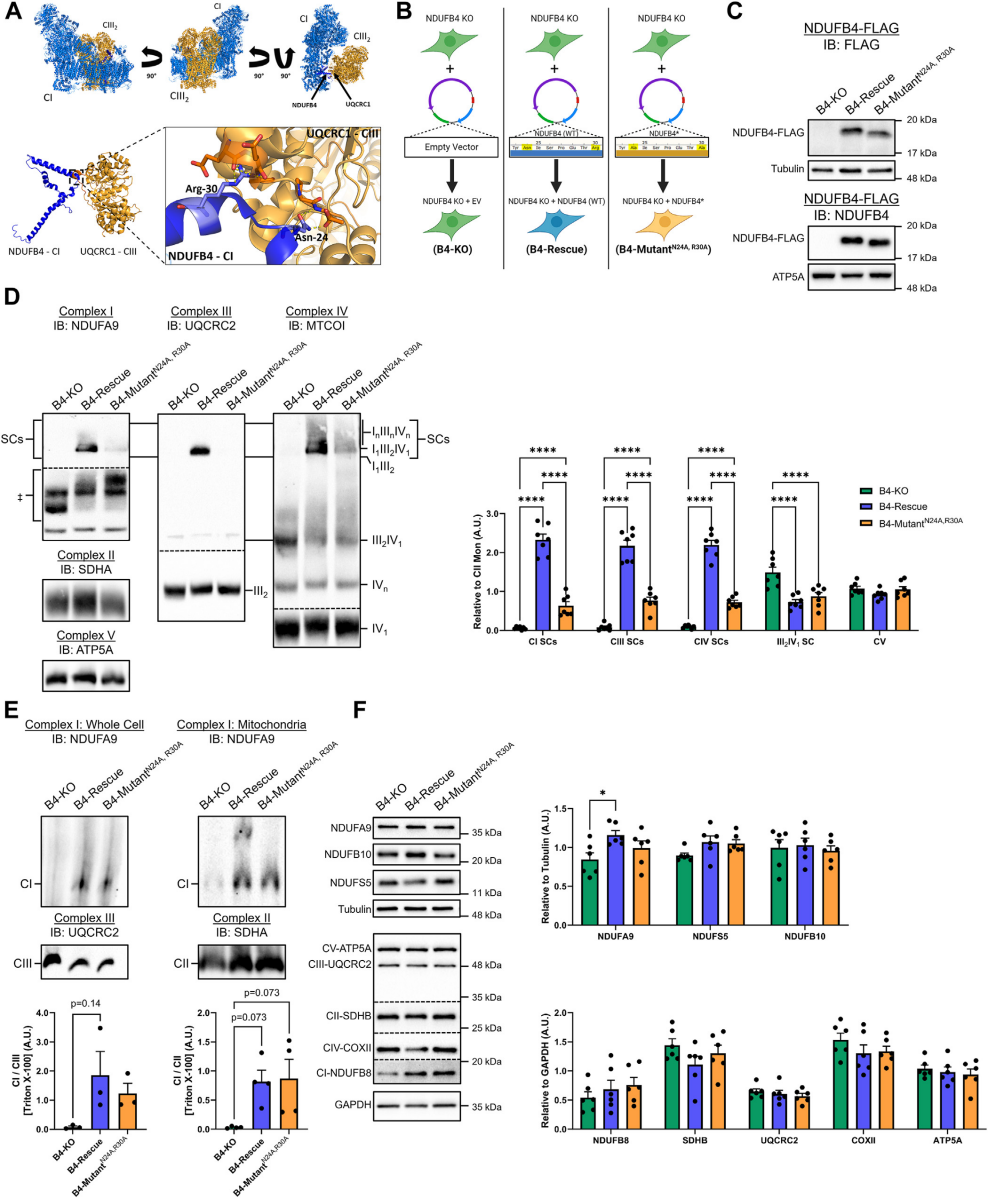
**N24A and R30A substitutions to CI accessory subunit NDUFB4 impair respirasome formation.***A*, analysis of respirasome crystal structure (PDB: 5XTH) identifies NDUFB4 residues N24 and R30 interacting with UQCRC1 of CIII_2_. *B*, schematic outline of stable cell lines generated through retroviral transduction, restoring FLAG-tagged NDUFB4, or a FLAG-tagged mutated NDUFB4 with N24A and R30A substitutions. Diagram created with BioRender.com. *C*, immunoblotting analysis using FLAG and NDUFB4 antibodies demonstrating similar expression of WT or N24A, R30A point mutation NDUFB4 subunits in the stable B4-rescue and B4-mutant^N24A, R30A^ cell lines. *D*, immunoblot analyses on digitonin-solubilized cells separated by BN-PAGE and incubated with the indicated primary antibodies against complex I, III, and IV related SCs relative to complex II, n = 7 biological replicates. ‡ denotes CI-containing subcomplexes. See also Fig. S2*A*. *E*, immunoblot analyses of cells and isolated mitochondria solubilized Triton X-100 and separated by BN-PAGE, n=3 to 4 biological replicates. *F*, immunoblots of CI subunits NDUFA9, NDUFB10, and NDUSF5 and steady-state levels of key OXPHOS complex subunits, n = 6 biological replicates. Values are means ± SEM. Comparisons between groups were determined using a one-way ANOVA with Tukey post hoc test. ∗*p* < 0.05, ∗∗*p* < 0.01, ∗∗∗*p* < 0.001, and ∗∗∗∗*p* < 0.0001. BN-PAGE, blue-native PAGE; OXPHOS, oxidative phosphorylation; SC, supercomplex.

**Figure S1:**
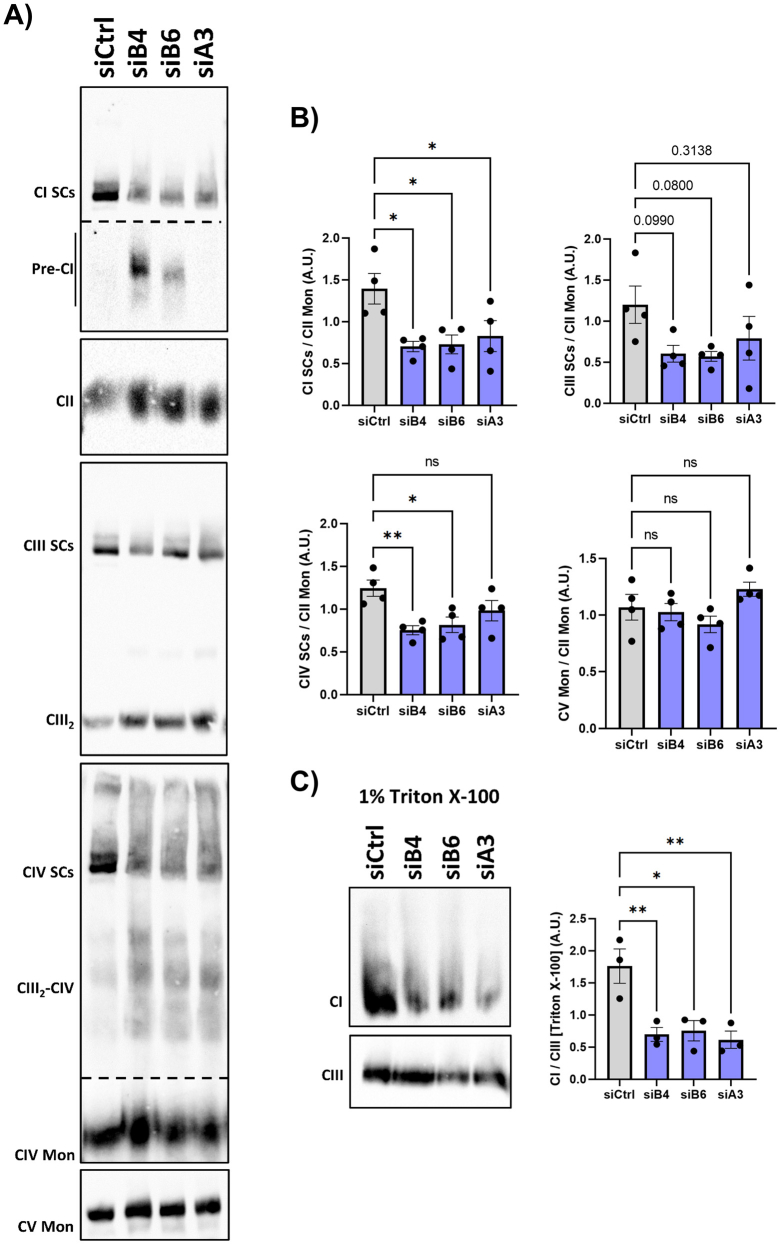
Loss of CI containing SCs and total CI following transient siRNA mediated knockdown of CI accessory subunits. *A*, HEK293t cells were transfected with siRNA for 48 hours prior to solubilization with 1.5% vol/wt digitonin, separation by BN-PAGE and immunoblotting with the indicated primary antibodies. siCtrl= negative control siRNA; siB4 = siRNA against CI subunit NDUFB4; siB6 = siRNA against CI subunit NDUFB6; siA3 = siRNA against CI subunit NDUFA3. *B*, levels of complex I (NDUFA9), III (UQCRC2) and IV (MTCOI)-containing respiratory SCs and complex V (ATP5A) relative to complex II (SDHA). Values are Means ± SEM, n = 4 from independent experiments. *C*, HEK293t cells were transfected with siRNA for 48 hours with prior to solubilization with 1% vol/vol triton X-100 detergent to disrupt all interactions between ETC complexes. Values are relative normalized intensities presented as Means ± SEM, n = 3 from independent experiments. Comparisons between groups were determined using a one-way ANOVA with Dunnet’s post-hoc test vs. siCtrl. ∗*p* < 0.05, ∗∗*p* < 0.01.

**Figure S2:**
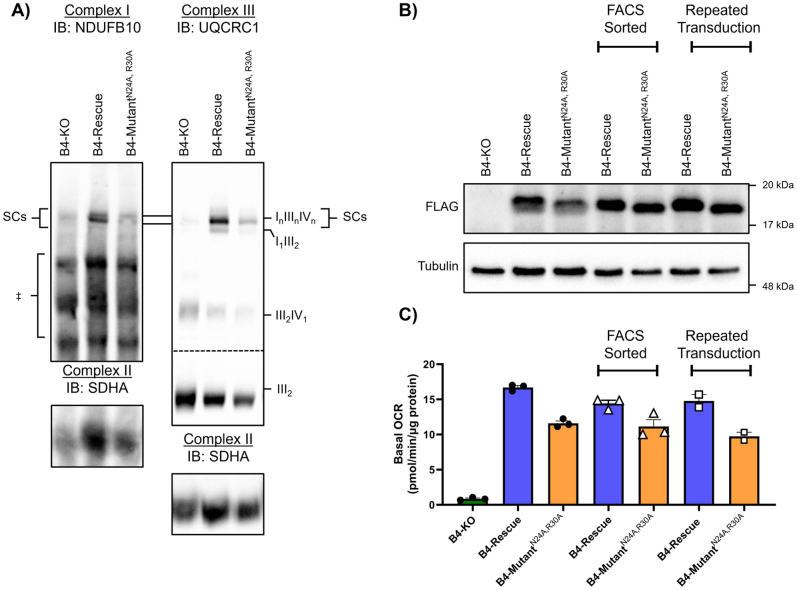
Cells harboring N24A and R30A substitutes for NDUFB4 display impaired respirasome formation. *A*, Immunoblot analyses on digitonin solubilized cells separated by BN-PAGE and incubated with primary antibodies against complex I (NDUFB10) and III (UQCRC1) related SCs relative to complex II, n = 3. ‡ Denotes CI-containing subcomplexes. *B* and *C*, Polyclonal stable cell lines were enriched by FACS sorting using the IRES-GFP. Separately, stable cell lines underwent an additional round of retroviral transduction to increase expression. *B*, FLAG immunoblot showing relative expression of WT or point mutated NDUFB4 following FACs sorting and repeated transductions. *C*, resting OCR of stable cell line populations following FACs sorting and repeated transductions. Means ± SEM, n =2-3 from independent experiments. Values are Means ± SEM. Comparisons between groups were determined using a one-way ANOVA with Tukey post-hoc test. ∗*p* < 0.05.

Further in-depth analyses of the human respirasome crystal structure data (PDB: 5XTH) (7) demonstrate that specific residues on the N terminus of NDUFB4 may form salt-bridging interactions with residues in the highly conserved loop (Y257-T266) of subunit UQCRC1 from CIII (27, 37) (Fig. 1, *A*). Importantly, complete loss of NDUFB4 results in incomplete CI assembly along with decreased expression of subunits in the P_D-b_ subassembly and N-module (25), and our attempt to knockdown NDUFB4 *via* siRNA similarly resulted in disruption of CI stability in addition to loss of respiratory SCs (Fig. S1). Therefore, we hypothesized that modification at polar residues Asn24 and Arg30 of NDUFB4 may disrupt CI super assembly while minimizing disturbance to CI integrity, allowing for an improved understanding of how NDUFB4 participates in respiratory SC formation. Using human embryonic kidney (HEK) 293T cells with a CRISPR/Cas9 NDUFB4-KO (B4-KO) (25), we rescued the stable expression of either WT NDUFB4 (B4-Rescue) or NDUFB4 with alanine substitutions at Asn24 and Arg30(B4-Mutant^N24A, R30A^). As a control, B4-KO cells were supplied with an empty vector (Fig. 1*B*). Subunits introduced by stable expression harbor a C-terminal FLAG tag to confirm relative expression levels (Fig. 1*C*). Next, we conducted blue-native PAGE (BN-PAGE) of digitonin-solubilized membrane proteins and performed immunoblot analysis of the Q module subunit, NDUFA9, which is located at the interface between the matrix and membrane arms (21, 26, 38). Consistent with previous findings (25), analyses of steady-state respiratory SCs demonstrated that ablation of NDUFB4 resulted in the loss of CI, CIII, and CIV containing respiratory SCs in the B4-KO cells (the I_1_III_2_IV_1_ respirasome) (Fig. 1*D*). In contrast, the readdition of WT NDUFB4 resulted in appreciable reassembly of the respirasome compared to B4-KO cells, as apparent through the restoration of CI, CIII, and CIV containing respiratory SCs (Fig. 1*D*). Levels of CI, CIII, and CIV containing respiratory SCs were lower in the B4-mutant^N24A, R30A^ cells than B4-rescue cells but higher than that of the B4-KO cells (*i.e.*, B4-rescue > B4-mutant^N24A, R30A^ > B4-KO). The B4-KO cells displayed elevated levels of the III_2_IV_1_ SC compared to both B4-rescue and B4-mutant^N24A, R30A^ cells, indicating a compensatory adaption in supramolecular organization of respiratory SCs (Fig. 1*D*). Respiratory SCs were similarly lower in both B4-KO and B4-mutant^N24A, R30A^ cells than the B4-rescue when immunoblotting for NDUFB10 and UQCRC1 subunits (Fig. S2*A*). We next confirmed the relative stability of CI by probing total CI levels through BN-PAGE with solubilization using Triton X-100 in whole cells and isolated mitochondria. Both B4-rescue and B4-mutant^N24A, R30A^ cell lines showed similar recovery of CI levels compared to B4-KO cells (Fig. 1*E*). To confirm whether impaired respirasome formation in B4-mutant^N24A, R30A^ cells could be attributable to decreased respiratory complex levels, we quantified the expression of key respiratory subunits with a focus on CI subunit levels. Expression of ETC subunits, including CI subunits, in the Q-module (NDUFA9), P_D_ module (NDUFB8 and NDUFB10) and P_P_ module (NDUFS5) were similar between B4-rescue and B4-mutant^N24A, R30A^ cells (Fig. 1*F*). Similarly, the expression of key ETC CI-V subunits was also comparable between groups (Fig. 1*F*). Taken together, selective point mutations N24A and R30A in NDUFB4 have allowed for respirasome disassembly with low impact on ETC complex stability.

### N24A and R30A mutations of NDUFB4 modify cellular bioenergetics

**Figure 2:**
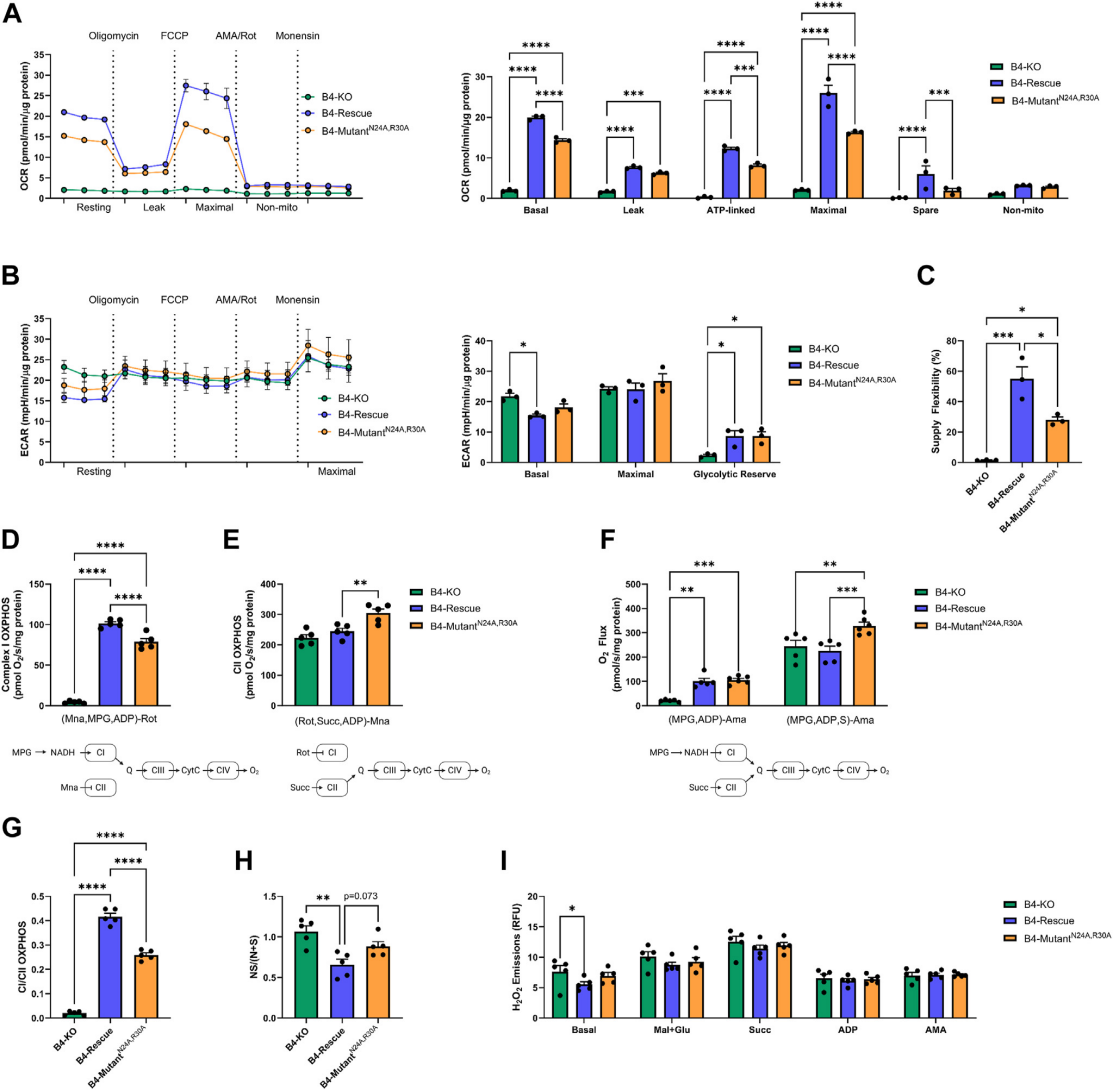
**N24A and R30A mutations of CI accessory subunit NDUFB4 modify cellular metabolism and substrate preference.***A*–*C*, Seahorse analyses of (*A*) oxygen consumption rate (OCR) and (*B*) extracellular acidification rate (ECAR) following sequential injections of oligomycin, FCCP, combined antimycin-A and rotenone, and then monensin. *C*, the calculated supply flexibility index, n = 3 from independent experiments. *D*–*H*, high-resolution respirometry of digitonin-permeabilized cells. *D*, rotenone (Rot)-sensitive CI-specific OXPHOS respiration measured in the presence of malonate (Mna), malate-pyruvate-glutamate (MPG), and ADP. *E*, malonate (Mna)-sensitive CII-specific OXPHOS respiration measured in the presence of rotenone (Rot), succinate (S), and ADP. *F*, costimulation of CI- and CII-linked OXPHOS using malate-pyruvate-glutamate (MPG), ADP, and succinate (S) corrected for nonmitochondrial oxygen consumption using antimycin-A (Ama). *G*, the ratio of CI-specific and CII-specific OXPHOS respiration. *H*, calculated comparisons of convergent electron flow, n = 5 to 6 from independent experiments. *I*, quantitative spectrofluorimetric analyses of ROS production following the addition of substrates in digitonin-permeabilized cells, n = 5 from independent experiments. Values are means ± SEM. Comparisons between groups were determined using a one-way ANOVA with Tukey post hoc test. ∗*p* < 0.05, ∗∗*p* < 0.01, ∗∗∗*p* < 0.001, and ∗∗∗∗*p* < 0.0001. FCCP, trifluoromethoxy carbonylcyanide phenylhydrazone; OXPHOS, oxidative phosphorylation.

**Figure S3:**
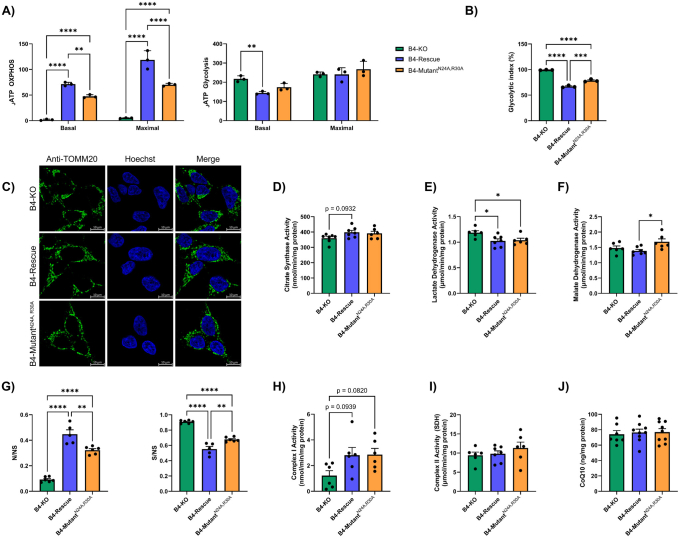
N24A and R30A mutations of CI accessory subunit NDUFB4 modify cellular metabolism and substrate preference. *A*, the rate of ATP formation from OXPHOS and glycolysis and OXPHOS, n = 3 (*B*) The glycolytic index, n = 3 from independent experiments. C, confocal microscopy of cells stained with TOMM20 and nuclei (Hoechst). *D*–F, enzymatic activity of (*D*) Citrate synthase, (*E*) Lactate dehydrogenase (LDH), and (*F*) Malate dehydrogenase, n = 6-7 biological replicates. G, contributions of N-linked respiration and S-linked respiration in the co-stimulated protocol, n = 5-6 from independent experiments. *H* and *I*, catalytic activity of (*H*) Complex I (CI) and (*I*) Complex II, n = 6-7 biological replicates. (*J*) Steady-state CoQ10 levels, n = 7-9 biological replicates. Values are Means ± SEM. Comparisons between groups were determined using a one-way ANOVA with Tukey post-hoc test. ∗*p* < 0.05, ∗∗*p* < 0.01, ∗∗∗*p* < 0.001, ∗∗∗∗*p* < 0.0001.

To understand the impact of respirasome disassembly on whole-cell bioenergetics, we performed Seahorse XF analyses to assess cellular respiration and glycolytic flux. In line with previous observations (39), B4-KO cells exhibited low basal, oligomycin-induced leak, maximal, and nonmitochondrial oxygen consumption rates (OCRs), consistent with the conclusion that ablation of NDUFB4 impairs cellular bioenergetics (Fig. 2*A*). Restoration of WT NDUFB4 and N24A, R30A-mutant NDUFB4 increased cellular respiratory capacity compared to B4-KO cells. Although recovery of nonmitochondrial respiration was similar, the resting, leak, and maximal OCRs were lower in B4-mutant^N24A, R30A^ cells than the B4-rescue cells, by 31%, 24%, and 40%, respectively. These deficits culminated to a 33% reduction in ATP-linked respiration (the difference between the basal respiration and leak respiration) in B4-mutant^N24A, R30A^ cells compared to B4-rescue cells. Attempts to increase stable expression of the modified NDUFB4 subunit through independent methods, including repeated transductions and fluorescence-activated cell sorting, did not improve resting respiration (Fig. S2, *B* and *C*). The overall suppression in endogenous respiratory capacity in the B4-mutant^N24A, R30A^ cells supports partial impairment of cellular bioenergetics when Asn24 and Arg30 residues on NDUFB4 cannot participate in SC formation. Analysis of extracellular acidification rates, a proxy measure of glycolysis, demonstrated that while B4-KO cells were highly glycolytic, both B4-rescue and B4-mutant^N24A, R30A^ cells had similar basal and reserve glycolytic capacity, suggesting that the N24A, R30A point mutations do not result in an increased reliance on glycolytic energy (Fig. 2*B*). Calculations of metabolic flexibility and ATP production using the Mookerjee *et al*. method (40) confirmed that B4-KO cells derived ∼99% of ATP from glycolysis (Fig. S3, *A* and *B*). ATP production from OXPHOS was restored in B4-rescue and B4-mutant^N24A, R30A^ cells, though oxidative ATP production was comparably lower in B4-mutant^N24A, R30A^ cells, resulting in a ∼12% higher glycolytic index (Fig. S3, *A* and *B*). Moreover, the B4-KO and B4-mutant^N24A, R30A^ cells had a reduced ability to respond to changes in ATP demand by switching the source of ATP supply between glycolytic and oxidative pathways compared to B4-rescue cells (Fig. 2*C*).

Immunocytochemical staining with TOMM20 indicated that the mitochondrial network was intact across cell lines (Fig. S3*C*), indicating that the decrements in oxidative metabolism in B4-KO and B4-mutant^N24A, R30A^ cells were not attributable to lower mitochondrial density. To confirm that mitochondrial content was maintained across cell lines, we quantified citrate synthase (CS) activity, a proxy measure of mitochondrial content (41). The activity of CS was similar between B4-rescue and B4-mutant^N24A, R30A^ cells, but tended to be slightly lower in B4-KO cells (*p* = 0.093) (Fig. S3*D*). Consistent with a shift in oxidative to glycolytic metabolism, B4-KO cells displayed higher rates of lactate dehydrogenase (LDH) activity than B4-rescue and B4-mutant^N24A, R30A^ cells (Fig. S3*E*). Moreover, malate dehydrogenase (MDH) activity was higher in the B4-mutant^N24A, R30A^ cells than both B4-KO and B4-rescue (Fig. S3*F*), which may be compensatory action to increase NAD reduction and tricarboxylic acid (TCA) cycle turnover.

To determine the respective contribution of CI and CII to the observed respiratory deficits, we independently stimulated the CI-linked (N-pathway) and CII-linked (S-pathway) ETC pathways in digitonin-permeabilized cells and assessed the impact using high-resolution respirometry. Rotenone-sensitive CI-specific OXPHOS was measured in the presence of malonate, a CII competitive inhibitor, following the addition of NADH-linked substrates (pyruvate, glutamate, malate) and ADP. Malonate-sensitive CII-specific OXPHOS was measured in the presence of rotenone, a CI inhibitor, following the addition of succinate and ADP. When assessing the capacity of the S-pathway, the addition of rotenone is particularly important to prevent the accumulation of oxaloacetate, a competitive inhibitor of CII, which is formed by the oxidation of malate by MDH in the mitochondrial matrix. Ablation of NDUFB4 severely impaired basal and CI-specific OXPHOS respiration in B4-KO cells, whereas CII-specific OXPHOS was functionally maintained (Fig. 2, *D* and *E*). Rescue of WT NDUFB4 increased CI-specific OXPHOS and did not alter CII-specific respiration compared to B4-KO cells. In contrast, while the expression of the NDUFB4 harboring N24A, R30A point mutations recovered CI-specific OXPHOS compared to B4-KO cells, CI-specific OXPHOS in B4-mutant^N24A, R30A^ cells remained lower than B4-rescue cells. (Fig. 2*D*). Moreover, CII-specific OXPHOS respiration was higher in B4-mutant^N24A, R30A^ cells than B4-rescue cells, suggesting a shift from CI- to CII-linked respiration following impaired respirasome formation (Fig. 2*E*). To determine full respiration capacities under the provision of saturating substrates, we then co-stimulated CI- and CII- linked pathways to support routine intracellular TCA cycle function and convergent electron flow from CI and CII to the CoQ pool. Despite impaired respirasome formation, B4-mutant^N24A, R30A^ cells display similar rates of CI OXPHOS and increased CI+CII-linked OXPHOS compared to B4-rescue cells in the absence of the inhibitors, rotenone and malonate (Fig. 2*F*). By following the sequential addition of substrates in the co-stimulation protocol, the proportion of CI-linked (N-pathway) or CII-linked (S-pathway) utilization can be estimated (42, 43). The B4-KO cells rely exclusively on the S-pathway for respiration, whereas B4-rescue cells restore N-pathway respiration (Figs. 2*G* and S3*G*). Consistent with the independently stimulated CI and CII respiration capacities, B4-Mutant^N24A, R30A^ cells shift away from the N-pathway and toward the S-pathway following impaired respirasome formation. (Figs. 2*G* and S3*G*). However, this was not due to an increase in catalytic capacity, as CII (SDH) activity was similar between cell lines (Fig. S3*I*). Similarly, CI catalytic capacity was comparable between B4-rescue and B4-mutant^N24A, R30A^ cells, though NDUFB4-KO cells also exhibited low CI activity (Fig. S3*H*).

To further elucidate potential substrate control of ETC oxidative function, we subsequently compared the additivity of respiratory capacity by summing the inhibitor-specific N-pathway and S-pathway respiratory capacities measured separately (N + S) to the respiratory capacity measured with combined substrates (NS). Complete additivity (NS = N+S) implies a lack of interaction between CI- and CII-linked pathways following convergent electron flow at CoQ, which may be due to individual substrate channeling and separation of dedicated CoQ pools for N-linked and S-linked electron flow. In contrast, incomplete additivity implies a partially antagonistic effect from simultaneous CI and CII convergent electron flow, which is typically observed in most cell types (44). Segmentation of N-linked and S-linked redox intermediates, including CoQ, is a proposed function of respiratory SCs that is a matter of active debate. When compared, B4-KO cells exhibited complete additivity (NS= (N+S)), likely due to minimal convergence of electrons from CI. In contrast, incomplete additivity was observed in B4-rescue cells (NS< (N+S)), indicating an interaction between redox intermediates from N- and S-pathways (Fig. 2*H*). The additivity in the B4-mutant^N24A, R30A^ cells tended to be more complete than that in B4-rescue cells (*p* = 0.073), suggesting there may be less interaction from CI and CII convergent electron flow. Of note, the steady-state levels of intracellular CoQ remained similar between all cell lines, suggesting that alterations to respiratory capacities are independent of CoQ production and turnover (Fig. S3*J*).

CI is a large source of mitochondrial ROS production (45), and CI ROS production is greatly increased when CI is chemically dissociated from SCs through N-dodecyl-β-d-maltoside (13). To determine if impaired CI SC formation affected ROS production in the B4-mutant^N24A, R30A^ cells, we used quantitative spectrofluorometry to measure H_2_O_2_ emission rates. H_2_O_2_ emissions were comparable between B4-rescue and B4-mutant^N24A, R30A^ cells, whereas basal H_2_O_2_ emissions rates were slightly higher in B4-KO cells prior to stimulation with ETC substrates, consistent with an overall respiratory defect (Fig. 2*I*).

### N24A and R30A mutations of NDUFB4 alter steady-state metabolomics

**Figure 3:**
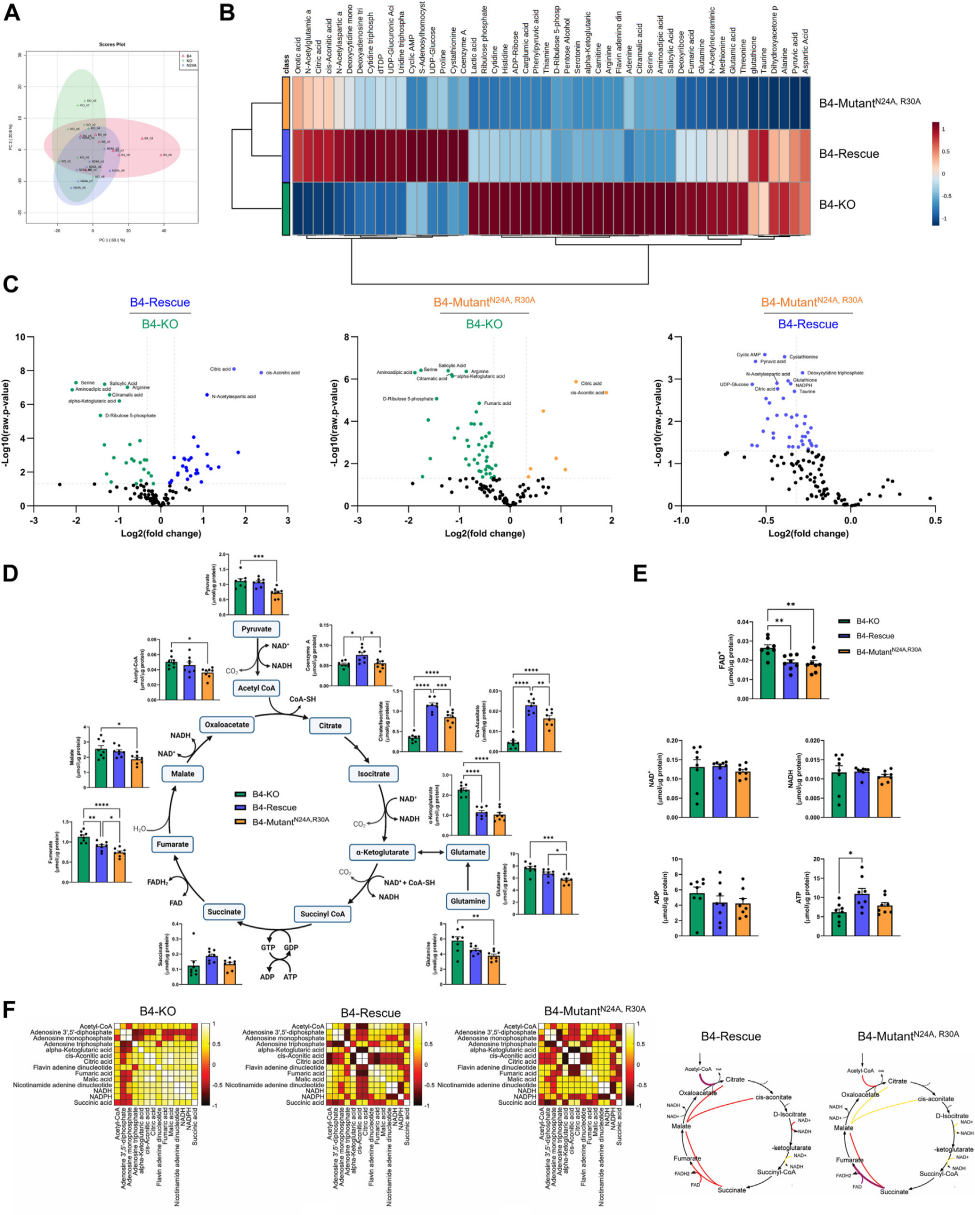
**N24A and R30A mutations of CI accessory subunit NDUFB4 alter steady-state metabolomics.** Metabolomic analyses of steady-state metabolite levels, n = 8 biological replicates. *A*, principal component analysis of independent samples. *B*, hierarchical clustering of the top 50 significant metabolites. *C*, *volcano plots* of metabolite profiles. *D* and *E*, quantitative analysis of individual metabolites relating to (*D*) the TCA cycle and (*E*) the electron transfer system. Diagrams created with BioRender.com. Values are means ± SEM. Comparisons between groups were determined using a one-way ANOVA with Tukey post hoc test. ∗*p* < 0.05, ∗∗*p* < 0.01, ∗∗∗*p* < 0.001, and ∗∗∗∗*p* < 0.0001. *F*, distance correlations of metabolites relating to the TCA cycle and electron transfer system. TCA, tricarboxylic acid.

**Figure S4:**
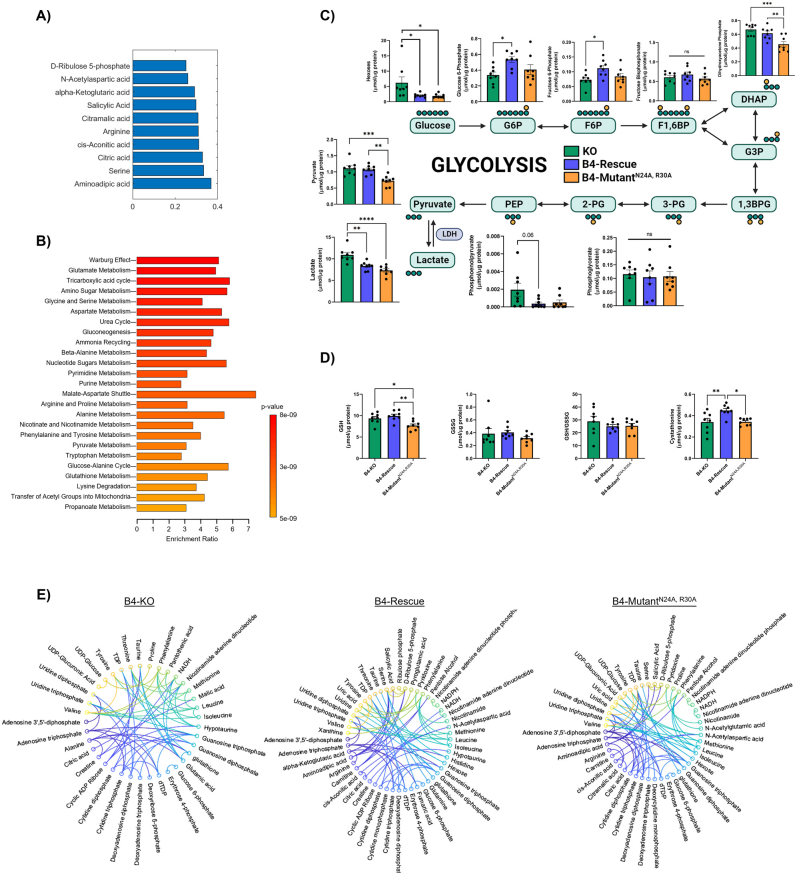
Steady-state metabolomic analyses. *A*, selection of the most significantly different features using the machine learning method ReliefF. *B*, Metabolite set enrichment analyses of the metabolites altered between groups. *C*, quantitative analysis of individual metabolites relating to glycolysis, n = 8 biological replicates. Values are Means ± SEM. Comparisons between groups were determined using a one-way ANOVA with Tukey post-hoc test. ∗*p* < 0.05, ∗∗*p* < 0.01, ∗∗∗*p* < 0.001, ∗∗∗∗*p* < 0.0001. *D*, quantitative analysis of individual redox metabolites, n = 8 biological replicates. *E*, comparison of distance correlation networks for metabolites. Shown are correlations >0.95, *p* < 0.001.

**Figure S5:**
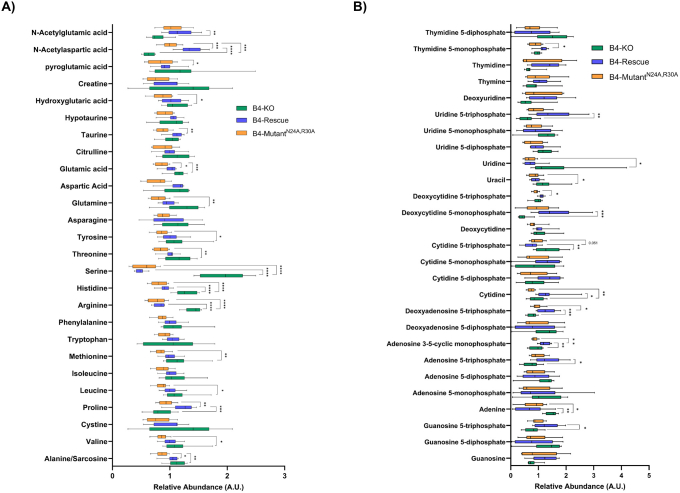
Steady-state metabolomic analyses of amino acids and nucleotide derivate metabolites. *A*, relative abundance of amino acid and (*B*) nucleotide derivate metabolites, n = 8 biological replicates. Summary box-whisker plots (whiskers displaying 10-90 percentile). Comparisons between groups were determined using a one-way ANOVA with Tukey post-hoc test. ∗*p* < 0.05, ∗∗*p* < 0.01, ∗∗∗*p* < 0.001, ∗∗∗∗*p* < 0.0001.

To determine whether substrate utilization was altered by respirasome deficiency, we performed quantitative metabolomics of 125 metabolites using ion-pairing LC-MS under steady-state conditions (Supporting information). Principal component and hierarchal clustering analyses of the global metabolite profiles revealed that B4-mutant^N24A, R30A^ cells are an intermediate between B4-rescue and B4-KO cells, though B4-mutant^N24A, R30A^ cells are more similar to B4-rescue cells (Fig. 3, *A* and *B*). After false discovery rate correction, we noted 51.2% differentially expressed metabolites. Interestingly, cells expressing NDUFB4 with the N24A, R30A point mutations had a global decrease in metabolite levels compared to cells rescued with WT NDUFB4 (Fig. 3*C*), consistent with the observed decrements in cellular metabolism. Next, we used the machine learning feature selection method, ReliefF, to identify individual metabolites that are important predictors of the metabolic phenotype. ReliefF identified and ranked metabolites in the TCA cycle (citric acid, *cis*-acontic acid, and alpha-ketoglutaric acid), aspartate metabolism (alpha-ketoglutaric acid, L-arginine, N-acetylaspartic acid) and the Warburg effect (citric acid, alpha-ketoglutaric acid, and D-ribulose 5-phosphate) as having high predictive scores (Fig. S4*A*). Consistent with these findings, metabolite set enrichment analyses of the altered metabolites highlighted significant differences in the Warburg effect, glutamate metabolism, and the TCA cycle between cell lines (Fig. S4*B*). Furthermore, B4-mutant^N24A, R30A^ cells exhibited blunted amino acid metabolism, and B4-KO cells had altered levels of nucleotide derivate metabolites (Fig. S5). Consistent with our Seahorse XF analyses of extracellular acidification rates (ECAR), comparisons of glycolytic metabolites indicate that the B4-KO cells rely heavily on glycolysis. Specifically, B4-KO cells had high concentrations of hexoses available for initial entry into the pathway, as well as high concentrations of end-stage glycolysis products lactate and pyruvate. Compared to B4-KO cells, glycolytic metabolite levels were lower in both the B4-rescue and B4-mutant^N24A, R30A^ cells, indicating the similar reliance on glycolysis between these cell lines. (Fig. S4*C*).

Individual analyses of TCA metabolites revealed low citrate/isocitrate and *cis*-aconitic acid levels in the B4-KO and B4-mutant^N24A, R30A^ cells compared to B4-rescue cells (Fig. 3*D*). Moreover, B4-mutant^N24A, R30A^ cells also exhibited decreased levels of pyruvate, glutamate, fumarate, and acetyl-CoA compared to B4-rescue cells. Of note, the concentration of succinate was maintained across cell lines, suggesting that CII-linked metabolism remains intact in B4-KO and B4-mutant^N24A, R30A^ (Fig. 3*D*). Despite these metabolites centering around NADH metabolism, the concentrations of NAD+ and NADH were maintained across cell lines (Fig. 3*E*). However, the calculated signed distance correlations between TCA metabolites support a change in metabolite preference, as there was a major difference between B4-rescue and B4-mutant^N24A, R30A^ in the correlation network of malate and citrate, malate, and *cis*-aconitate, and citrate/*cis*-aconitate and NADH (Figs. 3*F* and S4*E*). Moreover, reduced glutathione (GSH) levels were lower in B4-mutant^N24A, R30A^ cells than both B4-KO and B4-rescue cells; however, the ratio of GSH to oxidized GSSG, an indicator of oxidative stress, was maintained across cell lines (Fig. S4*D*).

## Discussion

To delineate the functional consequences of impaired respirasome assembly while avoiding changes to CI stability, we introduced selective modifications to residues in the CI accessory subunit NDUFB4 that are hypothesized to interact with CIII subunit UQCRC1 in CI-containing respiratory SCs. Our results show that N24A, R30A point mutations in NDUFB4 have a low impact on ETC complex stability but disrupt SC assembly. Using this N24A, R30A mutation model, we demonstrate that the loss of CI-containing respiratory SCs results in lower respiratory capacity and a compensatory increase in succinate-driven CII respiration, which together results in increased reliance on the S-pathway. Our integrated analysis of the steady-state metabolome identified key differences in NADH-generating substrate metabolites and a shift in substrate preference. Together, our data suggest that respiratory SCs may play a role in defining mitochondrial respiratory function.

Studies interrogating respirasome function often use chemical agents (13) that disrupt the MIM or genetic ablation of critical MIM proteins (46, 47). However, these experimental approaches likely interfere with delineating functional aspects that are specific to respiratory SCs by disrupting all pathways in which a protein operates. For example, most CI accessory subunit KOs do not display CI-containing SCs at a steady state, suggesting that accessory subunits are integral to CI formation and stability (25), including subunits at the interface between CI and CIII respiratory SCs. Indeed, we observed losses of total CI in addition to CI-SCs following transient knockdowns of NDUFB4 and NDUFB6, supporting the conclusion that accessory subunits are required for CI integrity. Introducing point mutations to a protein is less likely to result in a complete disruption of critical functions, which could allow for more intricate interpretations of specific types of protein–protein interactions (48). In contrast to total or partial ablation of NDUFB4, reintroducing a point mutated NDUFB4 subunit to B4-KO cells improved the integrity of CI while allowing for interrogation of respiratory SC function. In future, our point mutation strategy could be applied to other potential interactions between CI, CIII, and CIV subunit residues (26, 27, 28, 49) in studies of respirasome formation and function.

In addition to their role in CI assembly and stability (9, 10, 11), respiratory SCs are proposed to play a role in defining mitochondrial respiratory capacity. Our observations of decreased cellular respiration, low CI-specific OXPHOS, and low intracellular concentrations of CI substrates (malate, pyruvate, and glutamate) in B4-mutant^N24A, R30A^ cells support a role for respiratory SCs in enhancing CI cellular respiratory capacity. Importantly, we were unable to detect the deficiency in CI-specific respiratory capacity in B4-mutant^N24A, R30A^ cells in the absence of malonate, which prevents residual oxidation of succinate to fumarate, suggesting that appreciable S-pathway respiration can compensate and mask modest CI respiratory deficiencies. In line with our observations, mice with low levels of mitochondrial respirasomes induced by knock in mutations to critical amino acids on UQCRC1, including a residue proposed to interact with NDUFB4, demonstrated preserved mitochondrial respiratory chain capacity in isolated mitochondria when provided NADH-linked substrates in the absence of a CII inhibitor (50). The observed decreases in intact cellular respiration were potentially more apparent due to the exclusive provision of glucose, pyruvate, and glutamine largely stemming from N-pathway utilization, as succinate is not cell permeable. The increased CII OXPHOS respiratory capacity observed in B4-mutant^N24A, R30A^ cells indicates a compensatory adaptation in substrate utilization to maintain cellular energy metabolism. Consistent with these observations, under hypoxic conditions, MCF7 cells were found to have reduced levels of respirasomes along with decreased levels of citrate/isocitrate, *cis*-aconitate, and alpha-ketoglutarate, whereas concentrates of succinate and fumarate increased (51). Compensatory increases in CII-linked respiration are commonly observed in models of CI deficiency and under physiological conditions such as fasting, where respirasome levels and CI-linked respiration also decrease (15).

The costimulation of convergent electron flows through N- and S-pathways showed complete additivity for B4-KO cells, indicating that redox intermediates from the N-pathway and S-pathway do not interact, which is attributable to minimal input from the N-pathway. Rescue of SCs results in incomplete additivity (NS/N + S ratio drops), suggesting that there is greater mixing of redox intermediates. Restoring NDUFB4 limited the linked NS-pathway respiration, signifying crosstalk between the N- and S-pathway in the presence of SCs. These data provide evidence against a functional role of respiratory SCs in sequestering dedicated CoQ pools and substrate channeling, which is in line with kinetic and spectroscopic studies (52, 53). Instead, the incomplete additivity in B4-rescue cells signifies a negative feedback effect on the reciprocal pathway, potentially through the inhibitory actions of oxaloacetate on CII or substrate competition for CoQ. Studies on SCAF1 suggest that III_2_IV_1_ respiratory SCs can undergo reorganization to adapt to substrate availability through the actions of pyruvate dehydrogenase (43). Future studies should investigate the mechanisms of interplay between CI- and CII-linked respiratory pathways when respiratory SC levels are modulated.

It remains a matter of debate whether respiratory SCs can limit ROS production. Enhanced ROS production has been observed upon respiratory SC disassembly in bovine heart mitochondria (13), but was not apparent in liver or heart tissue from mice with impaired respiratory SC assembly due to UQCRC1 point mutations (50). In our model, NDUFB4 ablation and impaired respirasome assembly did not induce changes in ROS production, which is in line with previous observations (39). However, the lower levels of steady-state GSH indicate that antioxidant capacity may be decreased, and the increased reliance on succinate-driven respiration may drive reverse electron transport if membrane potential is high. Therefore, based on our data, we cannot discount that impaired respiratory SC assembly may have indirect pro-oxidant effects.

This study has several limitations. Our model investigating respiratory SC formation and function is limited to the scope of HEK293T cells. The validity of such amino acid substitutions to limit respirasome formation *in vivo* has yet to be determined. Moreover, the functional consequences following the disruption of interactions between complexes through subunit modifications have yet to be determined in a physiological setting. Though we demonstrate altered CI *versus* CII-linked respiratory capacities and limited whole-cell bioenergetics in our cell line model, the extent to which functional consequences follow respirasome impairment through this manner may vary. Lastly, we recognize that many other interactions between complexes I, III, and IV, other assembly factors (30, 35), and other important components critical to the structure and integrity of the MIM (46, 47, 54) contribute to respirasome formation. Future investigations could quantify the contribution of each of these factors to respiratory SC assembly and whether they are collectively or independently regulatory to physiological processes.

In summary, we show that CI accessory subunit NDUFB4 participates in respirasome assembly *via* Asn24 and Arg30 residue interactions. The disruption of CI-containing respiratory SCs leads to bioenergetic consequences, namely impaired CI respiration, which is compensated for by increases in CII respiration.

## Experimental procedures

### HEK293T cell line for siRNA-mediated knockdown experiments

HEK 293T cells were maintained at 5% CO_2_, 37 °C, cultured in Dulbecco’s modified Eagle's Medium (DMEM) (Gibco, 11995-065), and supplemented with 10% fetal bovine serum (FBS) (Wisent Bio Products) and 1% antimycotic-antibiotic (Gibco, 15240-062). Cell lines were subcultured 1:10 using trypsin-EDTA dissociation reagent when reaching ∼90% confluency.

### siRNA-mediated knockdown of mitochondrial respiratory complex subunits

500,000 unmodified HEK293T cells were plated 24 h prior to transfection in 60 mm cell culture plates, achieving ∼60% seeding density at the time of transfection. One hour prior to transfection, the cell culture media was replaced with high glucose DMEM supplemented with 10% FBS supplemented without antibiotics. Transfection was performed with siLentFect Lipid Reagent (Bio-Rad) according to the manufacturer’s protocol, with optimizations as indicated. The following siRNA were individually transfected per cell plate to a final concentration of 25 nM: NDUFB4 Silencer Select siRNA (Thermo Fisher Scientific, 4390824, siRNA ID: s224102), NDUFB6 Silencer Select siRNA (Thermo Fisher Scientific, 4392420, siRNA ID: s9366), NDUFA3 Silencer Select siRNA (Thermo Fisher Scientific, 4392420, siRNA ID: s9327), and Silencer Select Negative Control 1 siRNA (Thermo Fisher Scientific, 4390843). Ten microliters of siLentFect Lipid Reagent was used per 60 mm cell plate. Reagents were prepared separately in Opti-MEM reduced serum medium (Invitrogen) before being combined and added dropwise onto the cells. Forty-eight hours posttransfection, cells were harvested for downstream applications.

### Maintenance of NDUFB4-KO, B4-rescue, and B4-mutant^N24A, R30A^ cell lines

NDUFB4-KO HEK293T cells were a kind gift from Dr Mike Ryan (Monash University). Cell lines were maintained at 5% CO_2_, 37 °C, cultured in DMEM (Gibco, 11995-065), and supplemented with 50 μg/ml uridine (Sigma, U3003), 10% FBS (Wisent Bio Products), 1% Penicillin-Streptomycin (5000 U/ml) (Gibco, 15070-063), and 1× GlutaMAX (Gibco, 35050-061). Puromycin-resistant stable cell lines were maintained with 1 μg/ml puromycin dihydrochloride (Gibco, A11138-03). Media on NDUFB4-KO cells were replaced daily due to rapid acidification. Cell lines were subcultured 1:5 using trypsin-EDTA dissociation reagent when reaching ∼90% confluency.

### Generation of B4-rescue and B4-mutant^N24A, R30A^ stable cell lines

Stable cells were generated by retroviral transduction through subcloning into the MSCV-PIG vector (Addgene Plasmid #18751). Synthetic complementary DNA corresponding to human NDUFB4 (NM_004547.6), or modified with N24A and R30A substitutions were purchased from GenScript. Gibson assembly (New England Biolabs, E5510S) was performed to ligate complementary DNA constructs to XhoI/HPAI restriction digested and agarose gel purified MSCV-PIG vector. Resulting bacterial clones were verified to contain the insert using Sanger sequencing with the following primer: 5′-CCCTTGAASSTCCTCGTTCG-3′. Bacterial clones were amplified using the PureLink HiPure Plasmid Midiprep Kit (Invitrogen). Packaging plasmids pCMV-VSVG and pCMV-Gag-Pol (Cell biolabs, inc), and the backbone constructs were cotransfected into unmodified HEK293T cells using Lipofectamine LTX reagent (Thermo Fisher Scientific) in a 1:2:3 ratio. Forty-eight hours posttransfection, viral supernatant was harvested and filtered through a 0.45 μm filter. One milliliter of viral supernatant was combined with 400,000 NDUFB4-KO cells and 8 μg/ml hexadimethrine bromide (polybrene) and centrifuged at 800*g* for 1 h. Cells were then incubated with viral supernatant for 48 h, followed by replacement media supplemented with 2 μg/ml puromycin. To increase expression, cell lines required an additional round of retroviral transduction.

### BN-PAGE analysis of mitochondrial respiratory SCs

Whole-cell pellets and isolated mitochondria were assessed for respiratory SCs by BN-PAGE. Cells were resuspended in extraction buffer [50 mM imidazole/HCl pH 7, 50 mM NaCl, 5 mM 6-aminohexanoic acid, 1 mM EDTA] with either 1.5% w/v digitonin or 1% v/v Triton X-100, followed by gentle solubilization with agitation for 30 min, and then subsequently cleared by centrifugation for 30 min at 14,000*g*. The supernatant containing solubilized membrane proteins was mixed with glycerol (5% v/v final) and 5% Coomassie blue G-250 solution to achieve a 1:4 dye:digitonin ratio. Mitochondria were isolated as described previously and solubilized similarly with 1% v/v Triton X-100 (55). Samples were separated on 3 to 13% gradient gels and then transferred to nitrocellulose membranes. Membranes were probed using the following primary antibodies: NDUFA9 [20C11B11B11] (1:2000, Invitrogen, 459100), NDUFB10 (1:2000, Abcam, ab196019), SDHA [2E3GC12FB2AE2] (1:20,000, Invitrogen, 459200), UQCRC2 [13G12AF12BB11] (1:2000, Abcam, ab14745), UQCRC1 [16D10AD9AH5] (Invitrogen, 459140), MTCO1 [1D6E1A8] (1:2000, Thermo Fisher Scientific, 459600), ATP5A (1:50000, Abcam, ab14748). Membranes were washed three times for 5 min and probed with anti-mouse immunoglobulin G (IgG) horseradish peroxidase (HRP) secondary antibody in blocking buffer for 1 h at room temperature. Membranes were washed three times for 5 min in tris-buffered saline with 0.1% Tween-20 (TBS-T), and protein bands were visualized using the ChemiDoc MP Imaging System (Bio-Rad). Densitometry band analysis was performed using Image J software (https://imagej.net). Respiratory SC analysis was based on banding pattern as previously confirmed by 2D-BN-PAGE (6).

### Western blot analysis

Pelleted cells were resuspended in radioimmunoprecipitation lysis buffer (0.5 M Tris–HCl, pH 7.4, 1.5 M NaCl, 0.25% deoxycholic acid, 1% NP-40, 10 mM EDTA) supplemented with 1:500 protease inhibitor cocktail (Sigma, P8340). Mechanical lysis was performed through 10 strokes of a 28-gauge needle, followed by sample clearing at 10,000*g* for 10 min, 4 °C. Protein levels were quantified by the bicinchoninic acid (BCA) assay method using the Pierce BCA Protein Assay Kit (Thermo Fisher Scientific, 23225). Whole-cell protein lysate or isolated mitochondria was combined with 4× Laemmli Sample Buffer (62.5 mM Tris–HCl, pH 6.8, 10% glycerol, 1% SDS, 0.005% Bromophenol Blue) and 10% β-mercaptoethanol, followed by brief boiling for 5 min at 95 °C. Thirty micrograms of protein lysate was subjected to SDS-PAGE, followed by wet transfer to nitrocellulose membrane (Bio-Rad, 160112). Membranes were blocked in blocking buffer [5% skim milk in TBS-T] for 1 h at room temperature. Membranes were incubated with the indicated diluted primary antibodies in 1% bovine serum albumin (BSA) in TBS-T overnight at 4 °C under gentle agitation: 1:1000 ANTI-FLAG (Millipore, F7425), 1:10,000 Anti-α-Tubulin (Sigma-Aldrich, T6199), 1:2000 Total OXPHOS Human WB Antibody Cocktail (Abcam, ab110411), 1:2000 NDUFA9 [20C11B11B11] (Invitrogen, 459100), 1:2000 NDUFB10 (Abcam, ab196019), 1:2000 NDUSF5 (Abcam, ab188510), 1:1000 NDUFB4 (Abcam, ab110243), 1:5000 ATP5A (Abcam, ab14745), and 1:5000 GAPDH (Santa-Cruz, sc-47724). Membranes were washed five times for 5 min and probed with an anti-rabbit or anti-mouse IgG conjugated to HRP secondary antibody in blocking buffer for 1 h at room temperature. Protein bands were visualized using the ChemiDoc MP Imaging System (Bio-Rad). Densitometry band analysis was performed using Image J software.

### Immunocytochemistry

Cell lines were plated onto #1.5 thickness, 12 mm round glass coverslips (Thermo Fisher Scientific) coated with Matrigel at ∼20% confluency and allowed to adhere overnight. Coverslips were washed with PBS and subsequently fixed in 4% paraformaldehyde in PBS at room temperature for 15 min. Coverslips were washed with PBS and incubated with 1:200 TOM20 (ProteinTech Group, 11802-1-AP) antibody prepared in PBS and 1% BSA for 2.5 h. Coverslips were then washed three times with PBS and incubated with 10 μg/ml Hoechst33342 counterstain (Invitrogen, H3570) and 1:200 Donkey Anti-Rabbit IgG (H + L) Cy5 (Jackson ImmunoResearch, 711–175–152) for 2 h. Coverslips were mounted onto Fisherbrand Superfrost Plus Microscope Slides (Thermo Fisher Scientific) using Shandon Immu-Mount solution (Thermo Fisher Scientific). Images were acquired through a Zeiss LSM880 AxioObserver Z1 confocal microscope with AiryScan using a 63x 1.4 NA oil objective. Images were processed using ZenBlue 3.2 software (Zeiss; https://www.zeiss.com/microscopy/en/products/software/zeiss-zen.html).

### Enzyme activity assays

Enzyme activity assays were performed in cell homogenates for CS, LDH, MDH, and SDH activities and enriched mitochondrial fractions for complex I activity as previously described (56). Protein concentrations of cell homogenates and enriched mitochondria fractions were determined using the Pierce BCA Protein Assay Kit (Thermo Fisher Scientific, 23225). The rate of change of absorbance and path length of each well were determined using the BioTek Synergy Mx Microplate Reader (BioTek Instruments, Inc). Enzyme activities were calculated using extinction coefficients of 6.22 mM−1 cm^−1^ for LDH, MDH, and complex I; 13.6 mM−1 cm^−1^ for CS; and 19.1 mM−1 cm^−1^ for SDH.

### High-resolution respirometry of permeabilized cells

High-resolution respirometry of digitonin permeabilized stable cell lines was performed using the Oxygraph-2k system (OROBOROS Instruments). Cell lines were dissociated from culture flasks using Trypsin-EDTA and resuspended in MiR05 buffer (0.5 mM EGTA, 3 mM MgCl_2_-6H_2_O, 20 mM taurine, 10 mM K_2_HPO_4_, 20 mM Hepes, 110 mM sucrose, and 1 g/L BSA; pH 7.1). Two to three million cells were added to each measurement chamber, suspended in MiR05, and held at 37 °C. A total of three separate protocols were performed simultaneously in separate measurement chambers. The first protocol determining CI-specific respiration featured the following additions performed sequentially: 8.1 μM digitonin, 5 mM malonate, 2 mM malate, 5 mM pyruvate, 10 mM glutamate (CI leak respiration), 5 mM ADP (CI linked respiration), 0.5 μM rotenone (non-CI respiration), and 2.5 μM antimycin A (nonmitochondrial respiration). All respiration values were corrected by subtracting nonmitochondrial respiration values. CI-specific respiration was calculated by subtracting respiration values in the presence of rotenone. The second protocol determining CII-specific respiration featured the following additions performed sequentially: 8.1 μM digitonin, 0.5 μM rotenone, 0.2 mM succinate (CII leak respiration), 5 mM ADP (CII-linked respiration), 0.5 μM malonate (non-CII respiration), 2.5 μM antimycin A (nonmitochondrial respiration). All respiration values were corrected by subtracting nonmitochondrial respiration values. CII-specific respiration was calculated by subtracting respiration values in the presence of malonate. The third protocol determining CI+CII-linked respiration featured the following additions performed sequentially: 8.1 μM digitonin, 5 mM malonate, 2 mM malate, 5 mM pyruvate, 10 mM glutamate (CI leak respiration), 5 mM ADP (CI-linked respiration), 10 mM succinate (CI+CII-linked respiration), 2.5 μM oligomycin (CI+CII leak respiration), 0.5 μM titrations of trifluoromethoxy carbonylcyanide phenylhydrazone (uncoupled or maximal respiration), and 2.5 μM of antimycin A (nonmitochondrial respiration). Data is expressed per μg protein of each sample.

### Quantitative fluorometric mitochondrial ROS production assay

Mitochondrial H_2_O_2_ release was measured using a Hitachi F4500 spectrofluorometer in digitonin-permeabilized cells. Briefly, two million cells were resuspended in buffer Z (in mM: 110 K-MES, 35 KCl, one EGTA, three MgCl_2_, five K_2_HPO_4_, 0.5 mg/ml BSA; pH 7.3 at 37 °C) and incubated with 1.5 U ml^–1^ HRP and 1.5 μm Amplex red at 37 °C. Basal fluorescence readings were recorded prior to the addition of the following compounds: malate and glutamate (5 mM), succinate (5 mM), ADP (10 mM), and antimycin-A (8 μM). Rates of mitochondrial H_2_O_2_ production were calculated as change in fluorescence per minute.

### Seahorse analysis of OCRs and ECAR

OCR and ECAR were measured using the XFe96 Analyzer (Agilent Seahorse). 40,000 cells (experimentally optimized) were seeded onto 96-well XF96 culture plates coated with Matrigel. The following day, cell culture media was replaced with phenol red-free, sodium bicarbonate-free Seahorse XF Media pH 7.4 (DMEM, 25 mM D-glucose) supplemented with 4 mM L-glutamine and 1 mM sodium pyruvate on the day of the experiment. The XF96 culture plate was allowed to equilibrate at 37 °C in a non-CO_2_ incubator for 30 min prior to the start of the experiment. Following the acquisition of resting (basal) OCR and ECAR, cells were treated with the following compounds in order: 1.5 μM oligomycin to measure nonphosphorylating OCR, 1 μM trifluoromethoxy carbonylcyanide phenylhydrazone to measure uncoupled (maximal) OCR, 1.5 μM antimycin A and 1.5 μM rotenone to measure nonmitochondrial OCR, and 6.5 μM monensin to measure maximal glycolytic capacity. The amount of protein per well was determined by the BCA assay method using the Pierce BCA Protein Assay Kit (Thermo Fisher Scientific, 23225). Results are expressed as the average value from three independent experiments. Bioenergetic capacity and fuel flexibility characteristics were calculated as previously described (40).

### CoQ10 level determination by HPLC

HPLC analyses of quinone concentrations were conducted as previously described (57). In brief, cells were lysed in a radioimmunoprecipitation buffer and CoQ was extracted with a mixture of 28.5% ethanol and 71.5% hexane (vol/vol) by vigorously vortexing for 2 min. The polar and nonpolar phases were separated by centrifugation, and the upper organic layer was transferred to a clean tube, then was dried using a SpeedVac concentrator (Thermo Fisher Scientific) and kept at −80 °C. The samples were redissolved in a mixture of methanol and ethanol (7:3, vol/vol). Chromatography was performed using a reversed-phase C18 column (2.1 × 50 mm, 1.8 μm, Agilent m), eluted with mixture of 70% methanol and 30% ethanol (vol/vol) at 1.8 ml/min and detected at 275 nm (Agilent 1260 Infinity LC system). Retention times were confirmed using standards (Sigma-Aldrich). Protein content in the quinone extracts was determined by the BCA assay (Thermo Fisher Scientific) and used to normalize quinone levels.

### Metabolite extraction from cells and LC-MS analysis

Steady-state metabolomics were performed as previously described (58). Briefly, cells were seeded in 60 mm dishes to achieve 70 to 80% confluency after 48 h. Following 24 h of incubation with fresh media, cells were washed three times with ice cold 150 mM ammonium formate solution and quenched in an ice cold 50% MeOH/50% LC/MS water solution. The collected supernatants were quickly homogenized using a bead mill homogenizer at 4 °C (Fisherbrand Bead Mill 24 Homogenizer) in −20 °C equilibrated solution containing methanol, water, and acetonitrile (OmniSolv, Sigma). Homogenates were then incubated with a 2:1 dichloromethane:water solution on ice for 10 min. The polar and nonpolar phases were separated by centrifugation at 4000*g* for 10 min at 1 °C. The upper polar phase was dried using a refrigerated CentriVap Vacuum Concentrator at −4 °C (LabConco Corporation). Samples were resuspended in water and run on an Agilent 6470A tandem quadrupole mass spectrometer equipped with a 1290 Infinity II ultra HPLC (Agilent Technologies) utilizing the Metabolomics Dynamic multiple reaction monitoring Database and Method (Agilent), which uses an ion-pairing reverse phase chromatography. This method was further optimized for phosphate-containing metabolites with the addition of 5 μM InfinityLab deactivator (Agilent) to mobile phases A and B, which requires decreasing the backflush acetonitrile to 90%. Multiple reaction monitoring transitions were optimized using authentic standards and quality control samples. Metabolites were quantified by integrating the area under the curve of each compound using external standard calibration curves with Mass Hunter Quant (Agilent). No corrections for ion suppression or enhancement were performed. Metabolite concentrations are presented relative to the total protein of each sample.

### Data mining and network analysis

Signed distance correlation analysis was done using Matlab 2023a (Matworks Inc) and https://complimet.ca/sidco (59). ReliefF was used for feature selection (command relief running under Matlab) using Euclidean distances as a metric for feature similarity and the 100 nearest neighbors for weight assessment. Correlation analysis using the distance correlation calculations were performed using SIDCO (58) and using in-house routines developed in Matlab and *p*-values were calculated using Student's *t* cumulative distribution function. Fuzzy c-means clustering was performed in Matlab with further in-house routines developed for cluster analysis. Metabolite changes were determined using linear regression comparisons between the groups for each metabolite, as previously described (59).

### Statistical analysis

Statistical analyses were performed using Prism 9 software (GraphPad; https://www.graphpad.com). Statistical significance for siRNA-mediated knockdown experiments was performed using one-way ANOVA, followed by Dunnett’s post hoc method for comparison to siCtrl. Statistical significance between B4-KO, B4-rescue, and B4-mutant^N24A, R30A^ cell lines was determined using one-way ANOVA, followed by Tukey post hoc method for pairwise comparisons. Data are shown as means ± SEM. Statistical significance was accepted at *p* < 0.05, with *p* values listed or represented with the following denominations: ∗∗∗∗ *p* < 0.0001, ∗∗∗ *p* < 0.001, ∗∗ *p* < 0.01, and ∗ *p* < 0.05.

## Data availability

Data are available in the main text or the Supporting Information.

## Supporting information

This article contains supporting information.

## Conflict of interest

The authors declare that they have no conflicts of interest with the contents of this article.

## Supporting information

Parmar_NDUFB4_Metabolites
